# Crosstalk between septic shock and venous thromboembolism: a bioinformatics and immunoassay analysis

**DOI:** 10.3389/fcimb.2023.1235269

**Published:** 2023-11-09

**Authors:** Zhishu Li, Chaolan Wang, Xu Zhang, Xiaolin Xu, Meng Wang, Lixia Dong

**Affiliations:** ^1^ Department of Respiratory and Critical Care Medicine, Guangyuan Central Hospital, Guangyuan, China; ^2^ Department of Respiratory and Critical Care Medicine, Tianjin Medical University General Hospital, Tianjin, China; ^3^ Department of Emergency Medicine, Tianjin Medical University General Hospital, Tianjin, China; ^4^ School of Statistics, Renmin University of China, Bejing, China

**Keywords:** bioinformatics, inflammation, sepsis, venous thromboembolism, immunology

## Abstract

**Background:**

Herein, we applied bioinformatics methods to analyze the crosstalk between septic shock (SS) and venous thromboembolism (VTE), focusing on the correlation with immune infiltrating cells.

**Methods:**

Expression data were obtained from the Gene Expression Omnibus (GEO) database, including blood samples from SS patients (datasets GSE64457, GSE95233, and GSE57065) and VTE patients (GSE19151). We used the R package “limma” for differential expression analysis (*p* value<0.05,∣logFC∣≥1). Venn plots were generated to identify intersected differential genes between SS and VTE and conducted Gene Ontology (GO) and Kyoto Encyclopedia of Genes and Genomes (KEGG) Enrichment analysis. The protein-protein interaction (PPI) network of intersected genes was constructed by Cytoscape software. The xCell analysis identified immune cells with significant changes in VTE and SS and correlated them with significant molecular pathways of crosstalk. Finally, we validated the mRNA expression of crosstalk genes by qPCR, while Matrix Metalloprotein-9 (MMP-9) protein levels were assessed through Western blotting (WB) and Immunohistochemistry (IHC) in human umbilical vein endothelial cells (HUVECs) and mice.

**Results:**

In the present study, we conducted a comparison between 88 patients with septic shock and 55 control subjects. Additionally, we compared 70 patients with venous thromboembolism to 63 control subjects. Twelve intersected genes and their corresponding three important molecular pathways were obtained: Metabolic, Estrogen, and FOXO signaling pathways. The resulting PPI network has 194 nodes and 388 edges. The immune microenvironment analysis of the two diseases showed that the infiltration levels of M2 macrophages and Class-switched memory B cells were correlated with the enrichment scores of metabolic, estrogen, and FOXO signaling pathways. Finally, qPCR confirmed that the expression of MMP9, S100A12, ARG1, SLPI, and ANXA3 mRNA in the SS with VTE group was significantly elevated. WB and IHC experiments revealed that MMP9 protein was significantly elevated in the experimental group.

**Conclusion:**

Metabolic, estrogen, and FOXO pathways play important roles in both SS and VTE and are related to the immune cell microenvironment of M2 macrophages and Class-switched memory B cells. MMP9 shows promise as a biomarker for diagnosing sepsis with venous thrombosis and a potential molecular target for treating this patient population.

## 1 Introduction

Sepsis is a disease characterized by life-threatening organ dysfunction due to a dysregulated host response to infection (Shankar-Hari et al., 2016). Severe metabolic and circulatory abnormalities in sepsis can further deteriorate into septic shock, with mortality rates as high as 40%-50%, significantly higher than sepsis alone (Shankar-Hari et al., 2016). Patients with septic shock (SS) are at high risk for venous thromboembolism (VTE), which often affects both the upper and lower extremities and leads to deep vein thrombosis and pulmonary emboli. Several risk factors contribute to this increased risk, including severe systemic inflammatory response, immune imbalance, vascular endothelial injury, activation of thrombotic inflammatory pathways, disseminated intravascular coagulation and blood stasis (Boulaftali et al., 2013; Rayes et al., 2019; Huang et al., 2020). There is an increasing consensus suggesting that the systemic inflammatory environment in SS increases susceptibility to VTE (Kaplan et al., 2015; Bahloul et al., 2020). Although effective anticoagulation therapy for septic shock can reduce the fatality rate to a certain extent, it cannot effectively improve the condition and prevent the development of VTE (Kaplan et al., 2015; Sorenson and McCurdy, 2016). It is now understood that in SS, toxins can activate coagulation directly through chemical mediators on endothelial cells, monocytes, and neutrophils or indirectly due to proinflammatory cascades caused by immune imbalances, low complement function, and cytokine storm. The complex pathophysiological mechanisms of coagulation account for the limited efficacy of anticoagulant and anti-inflammatory treatment (Angus and van der Poll, 2013; Ramakrishnan and Detect-Dvt Investigators, 2017).

SS is a known risk factor for VTE, but the underlying mechanisms are not fully understood. Based on previous studies and the above description, we propose a scientific hypothesis that SS has a potential molecular mechanism to promote VTE and may be related to immune cells. Developing corresponding diagnostic and therapeutic biomarkers would enable more precise and effective management of these diseases, thereby improving the condition and reducing the mortality rate. Therefore, this study aimed to reveal the crosstalk between SS and VTE and the underlying molecular pathways and interactions between immune-infiltrating cells. Importantly, we validated the significant crosstalk genes in septic shock patients with VTE and MMP9 protein expression levels in a mouse model.

## 2 Materials and methods

### 2.1 Datasets

**Figure 1:**
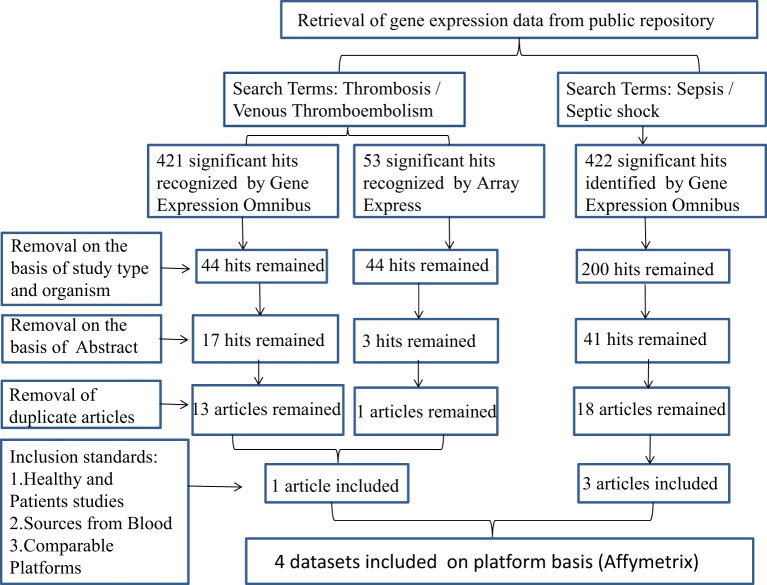
The flow chart describes the selection of microarray data analysis and the characteristics of individual studies included in the study. SS, septic shock; GEO, gene expression complex; VTE, venous thromboembolism.

We selected potential datasets from the Gene Expression Omnibus (GEO) database based on the following criteria: the dataset had to contain expression profiling by array data from human samples with sepsis or septic shock and thrombosis/venous thromboembolism, and the samples had to be from blood. We excluded datasets that were from other organisms or were not relevant to our research, as well as any duplicate articles. After screening, we obtained datasets GSE95233, GSE64457, GSE57065, and GSE19151 from the same platform (**Figure 1**). Expression data of SS and VTE were downloaded from the GEO database.

(https://www.ncbi.nlm.nih.gov/geo/query/acc.cgi?acc=GSE95233, accession number: GSE95233, https://www.ncbi.nlm.nih.gov/geo/query/acc.cgi?acc=GSE64457, accession number: GSE64457, https://www.ncbi.nlm.nih.gov/geo/query/acc.cgi?acc=GSE57065, accession number: GSE57065, https://www.ncbi.nlm.nih.gov/geo/query/acc.cgi?acc=GSE19151, accession number: GSE19151, access date: 1st April 2022).

### 2.2 Data preprocessing and differential expression analysis

We converted the probe IDs into gene symbols in accordance with the platform information for our chip data. If the same gene corresponded to multiple probes, the average value of the sample was used as the gene expression value of the sample. For SS, three datasets were integrated. We used the ComBat method to batch-correct the integrated data in R package SVA to reduce variability in sample batch combinations.

We used the R package “limma” for differential expression analysis of controls and patients with SS and VTE, respectively. To ensure the repeatability of the dataset, avoid batch differences, and obtain differential genes with higher repeatability, we screened the differences in the three original expression datasets of SS and then merged them. During the comparison between control and patients, a *p*-value < 0.05 and∣logFC∣≥ 1 was used as criteria for identifying a differentially expressed gene (DEG), with logFC ≥ 1 indicating an upregulated gene and logFC ≤ 1 indicating a downregulated gene. The FalseDiscovery Rate was used for correction.

### 2.3 Crosstalk gene analysis

We used Venn plots to identify potential crosstalk genes shared by SS and VTE by extracting differentially expressed genes in both sets. To avoid errors caused by data integration, we also used Venn plots to identify the overlap among the three sepsis datasets before identifying any crosstalk genes with VTE for subsequent analysis. To further analyze the function of crosstalk genes, the R package “clusterProfiler” was used for functional enrichment analysis (GO biological process and KEGG pathway). A *p*-value < 0.05 was statistically significant.

### 2.4 Crosstalk gene-related PPI network

In this study, we constructed a PPI network composed of crosstalk genes using the STRING online database tool (http://string-db.org, version 11.09) (Szklarczyk et al., 2017), which was designed to predict protein-protein interactions (PPIs). The results were then visualized as network models by Cytoscape (v3.7.2) (Shannon et al., 2003). Next, we used CytoHubba (Chin et al., 2014) to screen the top 12 key genes by degree and obtain their topological properties.

### 2.5 Immunology and stromal cell analysis

xCell is a genetic signature-based assay used to infer 64 immune and stromal cell types through extensive *in silico* analysis and can be compared to cytometric immunophenotyping, providing objective and reliable results (Aran et al., 2017). By applying xCell to microarray data and Wilcoxon ANOVA, estimated proportions of immune and stromal cell types were obtained for each SS and VTE sample. The cutoff value for cell analysis was *p* < 0.05. Cell types were divided into lymphoid, stroma, bone marrow, stem cells, etc. Venn plots were generated to obtain common cell types from different datasets.

### 2.6 Gene set enrichment analyses

Gene Set Variation Analysis (GSVA) is a method used to analyze KEGG-enriched pathways by estimating gene enrichment for changes in pathway activity in population samples, with thresholds set at enrichment score changes > 1.0 and *p* value < 0.05. Gene set enrichment analysis (GSEA) was performed by the GSEA desktop application to identify signaling pathways co-enriched in SS and VTE (Subramanian et al., 2005). Spearman correlation and linear regression analyses were performed on the correlation between immune cells and key KEGG pathways.

### 2.7 Quantitative real-time PCR

10 ml of venous blood was collected from patients and healthy volunteers. The clinical features of patients and healthy are shown in **Supplementary Data Table S1**. The RNA extraction from blood was conducted with TRIzol reagent (thermo). The cDNA synthesis was performed according to the manufacturer’s instructions (Vazyme, Nanjing, China) and real-time PCR analysis with Maxima SYBR Green QPCR Master Mix (Vazyme, Nanjing, China). RT-qPCR was performed using the API Prism 7900 H T Sequence Detection system according to the instruction of SYBER Green PCR Master Mix. Relative gene expression levels were calculated by the comparative CT method (2^-ΔΔCT^) (Schmittgen and Livak, 2008). The mRNA target gene expression levels were computed relative to the endogenous control GAPDH gene.

### 2.8 Cell culture

Single-donor lot human umbilical vein endothelial cells (HUVECs) (Zhejiang Meisen Cell Technology Co., Ltd, China, kindly donated by Prof.Jie Cao, Tianjin Medical University General Hospital, Tianjin) were cultured in 1640 (RPMI-1640; Gibco) medium with heat-inactivated 10% foetal bovine serum (FBS; Procell Life Science&Technology Co., Ltd. Wuhan, China) plus 100 mg/ml of streptomycin and penicillin in a 5% CO2 humidified atmosphere incubator at 37°C. LPS (1 μg/ml; Sigma-Aldrich, Cat# L2630) was used for HUVECs stimulation.

### 2.9 Mouse models

30 six-to-eight-week-old male wild-type (WT) C57BL/6 mice (18–22g) were purchased from Beijing Vital River Laboratory Animal Technology Co., Ltd. They were randomly divided into an experimental group and a control group. As previously reported, sepsis was induced by cecal ligation and puncture (CLP) surgery (Zhao et al., 2021). Mice were anesthetized with isoflurane. Under sterile conditions, a 2 cm long longitudinal incision was made in the midline of the abdomen, exposing the cecum. About 1cm from the tip of the cecum, the cecum was ligated with a 4-0 silk suture and punctured with a 20-gauge needle. The ligated cecum was squeezed, and a small portion of its contents was placed in the peritoneum and returned to the abdominal cavity. The incision was sutured with two layers of silk thread. Finally, 1 mL sterile saline (0.9%) was injected subcutaneously into the nape of the mice and warmed on a thermal blanket until recovery. The inferior vena cava was ligated 9h after sepsis modeling, and a model of sepsis with DVT was established. After isoflurane anesthesia, the inferior vena cava was exposed midline and ligated with 7-0 prolene (Ethicon, Inc, Somerville, NJ) at the inferior renal vena cava, resulting in hemostasis and eventually thrombosis (Obi et al., 2014). Finally, the fascia and skin were closed with a 5-0 Vicryl suture. The control group underwent the corresponding sham operation. The mice were sacrificed 6h after thrombosis, and plasma, venous wall, and thrombus samples were collected.

### 2.10 Western blotting

Cells were lysed with cell lysis buffer RIPA (Solarbio, Beijing, China, Cat# R0010) supplemented with protease inhibitor PMSF Solution (Solarbio, Beijing, China, Cat# P0100). The protein concentration in the extracts was measured using a BCA Protein Assay kit (Bioss, Beijing, China, Cat# C05-02001). Mouse inferior vena cava tissues were sonicated, and the supernatants were preserved after centrifugation at 12000×g for 15 min. Protein concentration was measured by a BCA assay kit (Beyotime). The proteins were separated by SDS-PAGE and transferred to PVDF membranes (Whatman, Germany). After blocking in 5% skim milk at room temperature (RT) for 1 hour, the membranes were incubated in primary antibody solution overnight at 4 °C. The primary antibodies, including MMP-9 (1:500; ProteinTech Group, Rosemont, IL, USA) and β-actin (1:2000; ProteinTech Group, Rosemont, IL, USA), were used to probe the target proteins. Goat polyclonal secondary antibodies to rabbit IgG-H&L (1:5000; Abcam, Alexa Fluor^®^ 488, UK) were used as secondary antibodies and were incubated with the membranes at RT for 2 h. Protein bands were visualized with an ECL detection kit (Affinity, KF001) and filmed using Tanon GIS digital image analytical system. Band density was quantified using Image J software.

### 2.11 Immunohistochemistry

Tissue sections embedded in paraffin and fixed with formalin were labeled with LSAB (Streptavidin-Biotin) and then dewaxed and rehydrated in xylene, ethanol, and TBS. Endogenous peroxidase (incubated in 3% H_2_O_2_ for 15 min) and endogenous biotin (Avidin/biotin blocking kit) were blocked. To obtain antigens, sections were incubated with protease K (DAKO, Carpinteria, CA) for 7 min, blocked with 1% BSA in PBS, and incubated with primary rabbit anti-MMP9 (1:100, ProteinTech) overnight in PBS. The sections were washed with 0.1% BSA and PBS and processed with Biotin Sheep Anti-Rabbit secondary antibody (Abcam) and AEC peroxidase substrate kit (Vector Laboratories) according to the manufacturer’s instructions. Those sections were then scanned by the Pannoramic DESK slide scanner (3DHISTECH, Hungary). The staining intensity was analyzed by Image-ProPlus 6.0 (Media Cybernetics, Inc., Bethesda, MD).

### 2.12 Statistics

Data are reported as arithmetic means ± Standard Error of the Mean (SEM). Unpaired Student’s t-tests were used to compare two groups of data with GraphPad Prism 9.5.1. *P* < 0.05 indicated statistically significant in all analyses.

## 3 Results

**Figure 2:**
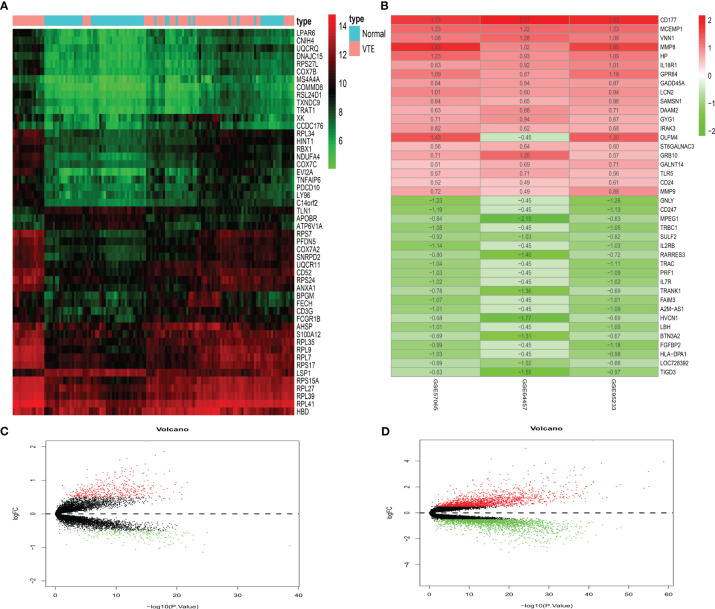
Differentially expressed genes. **(A)** Displays a heatmap of the top 50 significantly differentially expressed genes in VTE. **(B)** Displays the top 20 up-regulated and down-regulated genes in the three datasets of SS. **(C, D)** Show the VTE and SS volcano plots, respectively. Red means up-regulation and green means down-regulation.

We performed differential expression analysis on SS and VTE datasets and used R to generate the cluster heatmaps and volcano plots. A total of 626 DEGs (429 upregulated and 197 downregulated) were identified in the GSE19151 dataset (**Figure 2A, C**). In addition, the intersection of the three datasets of SS yielded 148 DEGs (123 upregulated and 25 downregulated) (**Figure 2B, D**). It can be seen that there are significantly more up-regulated genes than down-regulated genes in the two diseases. In order to further characterize these genes and DEGs, we will further analyze them.

**Figure 3:**
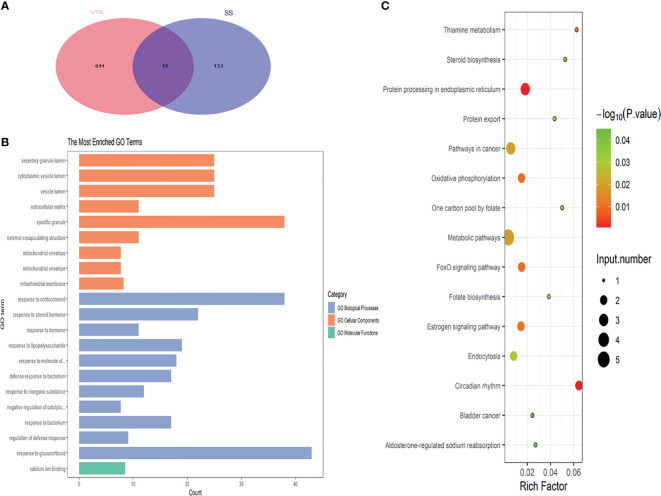
**(A)** Displays the Venn plot of the crosstalk gene in the four datasets. **(B)** Shows the biological process of crosstalk gene enrichment. **(C)** Presents the key KEGG pathway for crosstalk gene regulation.

DEGs between SS and VTE were identified by their intersection (**Figure 3A**). These genes are TRANK1, UGCG, METTL9, MS4A4A, CNIH4, S100A12, ALPL, RNASE2, BCL2A1, SLPI, FKBP5, MMP9, ARG1, ANXA3 and SAMSN1. To further analyze the types and functions of the above-mentioned crosstalk genes and the changes in the expression values of these genes in different types of samples, the R package “clusterProfiler” was used to perform functional enrichment analysis, with a *p*-value < 0.05 considered statistically significant. The results showed that their biological processes mainly involved response to corticosteroids and response to glucocorticoids. These enriched biological processes play an important role in the evolution of the pathophysiological mechanism of sepsis and VTE, and are also important biological characteristics that we pay attention to. Significantly enriched molecular pathways mainly involved metabolic pathways, including estrogen and FOXO signaling pathways, and pathways in cancer (**Figure 3B, C**).

**Figure 4:**
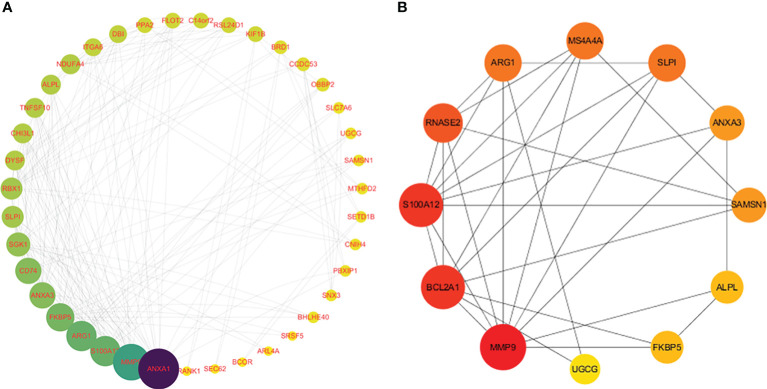
Analysis of protein-protein interaction (PPI) networks and related functional networks. Larger values are dark blue, while smaller values are yellow. A larger node degree value is indicated by a larger diameter of the point. **(A)** 39 genes of the PPI network. **(B)** 12 genes of the PPI network.

To further explore the underlying mechanisms of crosstalk genes, we utilized Cytoscape to construct a PPI network based on the STRING database. After merging the three SS datasets and removing genes that were not intersected, 39 crosstalk genes were identified. The obtained PPI network had 194 nodes and 388 edges in **Figure 4A**. A PPI network was constructed for the differential genes common to the four datasets. The obtained 12 genes of the network had 36 nodes and 62 edges in **Figure 4B**. The topological properties of the 12 genes are shown in **Table 1**. Through the results of DEGs and PPI obtained by the above two methods, it can be seen that MMP9 is the core hub gene in the two figures, which may play an important role in SS and VTE.

**Table 1:** The topological properties of the 12 genes.

Gene	Label	Degree	Average Shortet Path Length	Betweenness Centrality	Closeness Centrality	Topological Coefficient
MMP9	Cross	9	1.181818	0.226364	0.846154	0.474747
SlOOA12	Cross	8	1.272727	0.103636	0.785714	0.522727
BCL2Al	Cross	8	1.272727	0.196970	0.785714	0.512500
RNASE2	Cross	6	1.454545	0.026364	0.687500	0.590909
SLPI	Cross	5	1.545455	0.021818	0.647059	0.618182
MS4A4A	Cross	5	1.545455	0.007576	0.647059	0.636364
ARGl	Cross	5	1.545455	0.056061	0.647059	0.580000
SAMSN1	Cross	4	1.727273	0.000000	0.578947	0.675000
ANXA3	Cross	4	1.727273	0.021212	0.578947	0.625000
ALPL	Cross	3	1.909091	0.009091	0.523810	0.592593
FKBP5	Cross	3	1.727273	0.018182	0.578947	0.606061
UGCG	Cross	2	2.000000	0.003636	0.500000	0.785714

**Figure 5:**
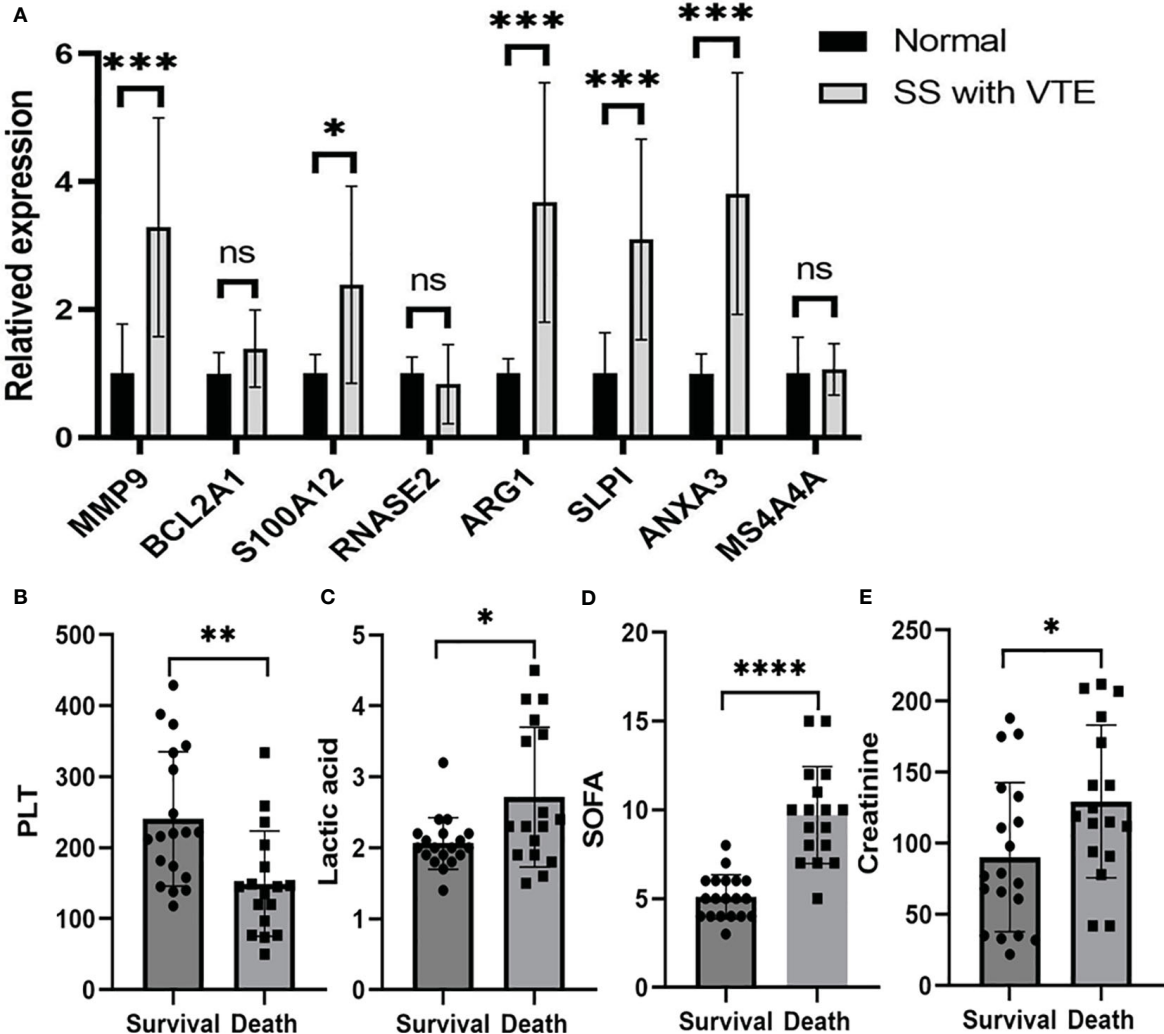
Validation of mRNA expression of selected genes in peripheral blood of patients with sepsis and VTE and healthy controls. **(A)** Shows the MMP9, BCL2A1, S100A12, RNASE2, ARG1, MS4A4A, SLPI and ANXA3 mRNA expression in patients and control group. Comparisons of the PLT **(B)**, lactic acid **(C)**, SOFA **(D)** and creatinine **(E)** of the patients with SS and VTE between the survival group and the death group. The data were expressed as mean ± SEM, *P < 0.05, **P < 0.01, ***P < 0.001, ****P < 0.0001, ns, no significance.

We used Real-Time PCR to analyze the expression levels of intersected genes. The study included 12 patients with SS and VTE and 10 healthy individuals as controls. We focused on the top 8 genes of the intersected genes list and confirmed their expression levels. The primer sequences for these genes are presented in **Supplementary Data Table S2**. The results indicated that MMP9, S100A12, ARG1, SLPI, and ANXA3 exhibited significantly higher expression levels than those observed in healthy controls (**Figure 5A**). These results are basically consistent with previous gene sequencing results and research reports. However, the expression of these genes in patients with sepsis alone and VTE alone and their specific functions need to be further studied by experiments. In order to track the prognosis of patients with SS and VTE, we retrospectively analyzed 36 patients with SS and VTE, including the above patients. Their general clinical characteristics were included in **Supplementary Data Table S3**. Patients with SS and VTE were divided into survival and deceased groups based on their prognosis within 28 days. Through comparative analysis, we found that there were no significant differences in coagulation function (D-dimer, Prothrombin time, International normalized ratio, Activated partial thromboplastin time, Fibrinogen, Thrombin time) and inflammatory response marker (IL-6, PCT) between the two groups. Interestingly, we found significant differences in PLT, liver function-related lactic acid, sequential organ failure assessment (SOFA), and kidney function-related creatinine between the two groups, which were associated with 28-day prognosis in patients with SS and VTE (**Figures 5B–E**). Next, a multivariate Cox regression analysis was performed on the above-mentioned indicators with statistical significances between the two groups. The result shows that the hazard ratio (HR) of PLT was 0.99 and the 95% confidence interval (95% CI) was [0.98–1.00] and the HR of SOFA was 1.54 with a 95% CI of [1.18–2.01]. The results suggested that PLT and SOFA are the independent risk factors that affected patient prognosis.

**Figure 6:**
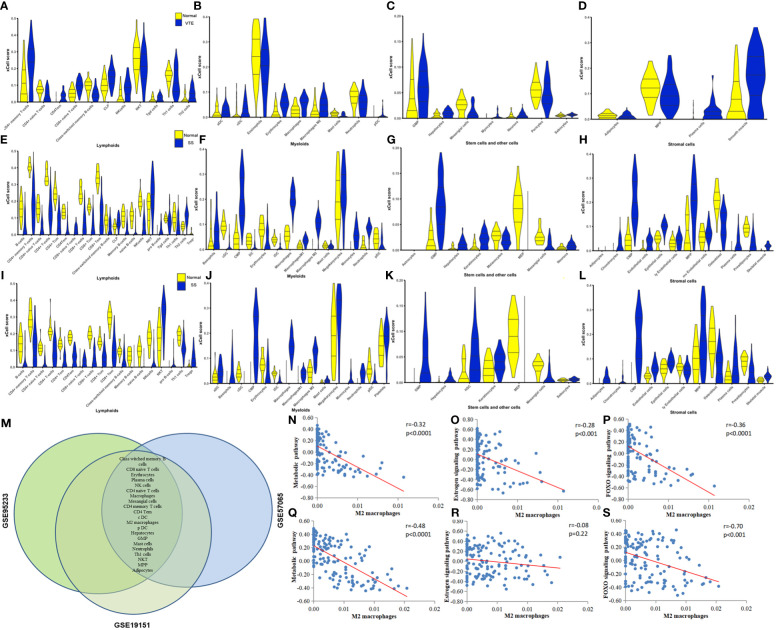
**(A-D)** Violin plots of significantly different lymphoid cells, myeloid cells, stem and other cells, and stromal cells in the GSE19151 dataset. **(E-H)** Violin plots of significantly different lymphoid cells, myeloid cells, stem and other cells, and stromal cells in the GSE95233 dataset. **(I-L)** Violin plots of significantly different lymphoid cells, myeloid cells, stem and other cells, and stromal cells in the GSE57065 dataset. **(M)** Differential cell types common to the GSE19151, GSE95233, and GSE57065 datasets. **(N-P)** Correlation between key pathways and immune cells. The correlation between M2 macrophages and metabolic pathways, estrogen signaling pathway, and FOXO signaling pathway in dataset VTE is demonstrated. **(Q-S)** The correlation between M2 macrophages and metabolic pathways, estrogen signaling pathway, and FOXO signaling pathway is demonstrated in the datasets of SS.

Immune and stromal cell deconvolution analyses. To identify immune cell types involved in the disease response to SS and VTE, we used xCell, which uses extensive gene expression data to generate cell types for enrichment scores. The 31 cell types significantly different from the normal group in the GSE19151 dataset are shown in **Figure 6A-D**. 54 cell types significantly differed from the normal group in the GSE95233 dataset (**Figure 6E-H**). 51 cell types significantly differed from the normal group in the GSE57065 dataset (**Figure 6I-L**). For example, we find that the scores of CD4+ Tem, pDC, Macrophages, Th2 cells, NK cells in VTE group were significantly increased in GSE19151 dataset. The scores of Th1 cells, NK cells, Memory B-cells, Macrophages, iDC, CD8+Tem, CD8+Tcm, CD8+T-cells, CD4+Tem, CD4+Tcm, CD4+ T-cells, B-cells in SS group were significantly decreased except Monocytes. These results of immune-infiltrating cells reflect the difference of immune microenvironment between the disease group and the control group to some extent, and reveal the behavior of various immune cells in different disease backgrounds. The common differential cell types were screened by generating a Venn plot (**Figure 6M**). (Note: Due to the small number of cases in the GSE64457 dataset (9 patients and 8 cases), xCell analysis could not be completed, and the immune-infiltrating cell types in this dataset were not presented). Next, the correlation of common signaling pathways with immune cells was analyzed. We first performed GSEA and GSVA to screen the biological differences between SS, VTE, and normal tissues. GSVA results showed that metabolic, estrogen, and FOXO signaling pathways were significantly enriched in both disease datasets and obtained enrichment scores. To evaluate the correlation between the aforementioned signaling pathways and immune cell infiltration, we conducted a Spearman correlation analysis to assess the relationship between the pathway enrichment scores and the proportions of immune cell infiltration. We found correlations between M2 macrophages, Class-switched memory B cells, and the above three pathways, which were verified in the SS and VTE datasets. The scatter plots depicting the correlations between M2 macrophages and these pathways are shown in **Figure 6N-S**. Further details on the correlation of Class-switched memory B cells with the above three pathways are provided in **Supplementary Figure S1**.

**Figure 7:**
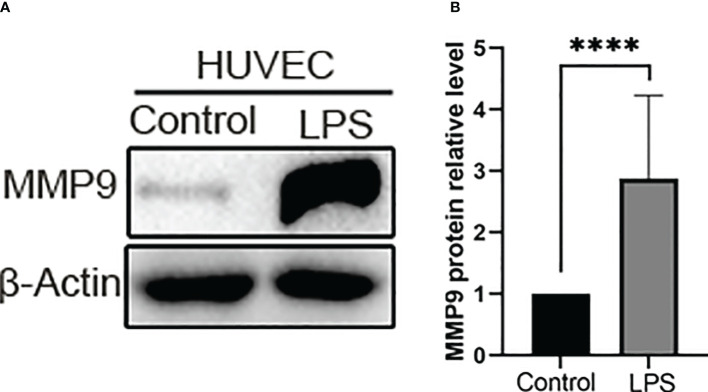
The expression of MMP-9 increased in the LPS group. **(A)** Representative western blot images of MMP-9 protein in the control group and LPS group. Western blotting other images data are provided in **Supplementary Figure S2**. **(B)** Quantitative analysis of MMP-9 expression in the control group and LPS group. ****P < 0.0001.

Venn plot and PPI analysis of DEGs suggest that MMP9, as a common differential gene of SS and VTE, is also a key gene of PPI. We speculate that MMP9 may be an important link in the interaction between sepsis and VTE, and an important molecule that worsens the disease and amplifies the clinical effect when sepsis and VTE are comorbidities. In terms of mechanism, MMP9 may play an important role in promoting the formation of VTE in sepsis. However, in the context of various pathogenic microorganisms, different immune microenvironments, different organs and tissues, and different clinical syndrome phenotypes of sepsis and VTE, the specific function and mechanism of MMP9 are still poorly understood. Previous studies have found that MMP-9 responds to various inflammatory mediators and is released rapidly in the body circulation, and MMP9 deficiency can prevent the death of LPS-induced sepsis models by alleviating harmful systemic inflammatory responses generated by the host (Dubois et al., 2002). However, the expression of MMP9 *in vitro* is not clear. Therefore, in order to evaluate the role and influence of *in vitro* sepsis on vascular endothelium, we next examined the expression level of MMP9 protein after LPS stimulation of HUVECs. LPS significantly increased MMP9 expression in HUVECs for 6 h (*p* < 0.0001, **Figure 7B**).

**Figure 8:**
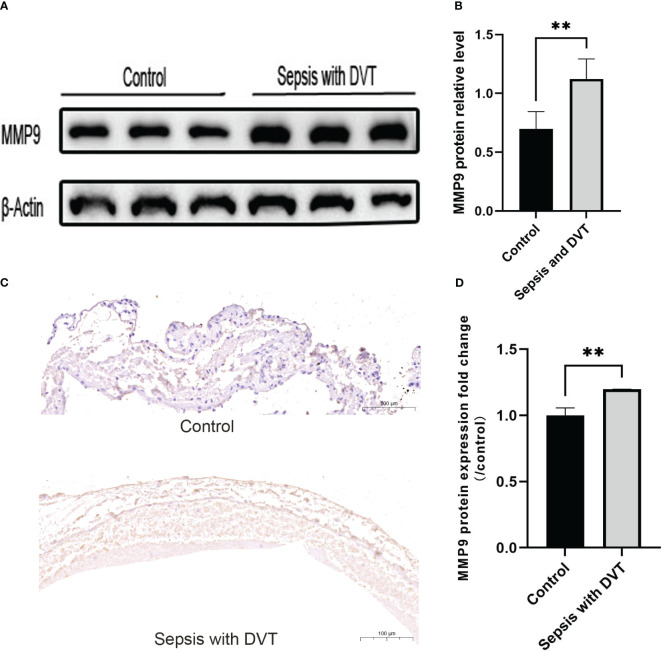
The expression of MMP-9 increased in the experimental group. **(A)** Representative western blot images of MMP-9 protein in the control group and sepsis with DVT group. **(B)** Quantitative analysis of MMP-9 expression in the control group and sepsis with DVT group. Immunohistochemistry was used to detect the expression of MMP9 in the inferior vena cava of mice in the sham operation group and sepsis with thrombus group. **(C)** Compared with the sham operation group, the experimental group had stronger immune reactivity (scale bar = 100 μm); **(D)** Compared with the control group, the expression level of MMP9 in the experimental group was significantly increased. Data are expressed as mean ± SEM. *P < 0.05, **P < 0.01, n=3–5.

MMP9 was highly expressed in the veins of mice with sepsis and DVT. MMP is a family of enzymes involved in the degradation of extracellular matrix components such as collagen, fibronectin, and laminin and is an important regulatory enzyme in proinflammatory and anti-inflammatory pathways (Obi et al., 2014). While it has been established that MMP9 activity in lymph fluid (Pan et al., 2014), lung tissue (Zheng et al., 2021), and liver (Biron-Pain and St-Pierre, 2012) of CLP mice is significantly increased, the expression of MMP9 in the inferior vena cava in CLP-induced DVT model remains unclear. Therefore, we studied the expression of MMP9 in mice with sepsis with DVT. The protein expression of MMP9 in mice with sepsis and DVT was significantly higher than in the control group (**Figure 8A, B**), which was further validated during the IHC assay conducted on venous wall samples (**Figure 8C, D**). These results suggest that MMP9 is involved in the pathological process of sepsis complicated with DVT.

## 4 Discussion

As a proinflammatory mediator, MMP-9 regulates growth factors, chemokines, cytokines, apoptosis signaling factors, and immune responses. Current evidence suggests that MMP-9 plays an important role in the pathogenesis of sepsis shock and is associated with various signaling pathways, such as nuclear factor κB (NF-κB) (Piirilä et al., 2010; Lauhio et al., 2011). In SS, MMP-9 demonstrates a significant early association with blood glucose levels. Elevated MMP-9 levels can lead to a reduction in IL-8 and intercellular cell adhesion molecule-1 (Sachwani et al., 2016). These findings suggest that targeting the upregulation of MMP-9 could be a potential therapeutic strategy for SS. It has been established that the anticoagulant edoxaban can regulate thrombosis factors, improve venous lesions, and inhibit venous thrombosis through MMP9-induced PI3K/AKT signaling pathway (Song et al., 2017). However, current reports on whether MMP-9 can predict the mortality of sepsis patients are inconsistent, which may be attributed to the heterogeneity of disease severity and research methods employed across studies. Few studies have hitherto assessed the role of MMP9 in thrombosis and its specific mechanism in increased susceptibility to VTE formation in SS patients. Moreover, the interaction between MMP9 and other key proteins in sepsis formation has also been poorly explored. Accordingly, further study of MMP9 can provide a comprehensive understanding of its role in sepsis with VTE.

Through differential gene screening and protein-protein interaction network analysis, we selected the top 8 genes for verification in patients. Significant differences in MMP9, S100A12, ARG1, SLPI, and ANXA3 expression were observed, consistent with the database results. Moreover, these results were consistent with previous studies on sepsis patients (Grobmyer et al., 2000; Uhel et al., 2017; Toufiq et al., 2020). Unfortunately, these differentially expressed biomarkers are not widely used in the clinic. Further investigation is warranted to determine whether combining biomarkers with commonly used inflammatory markers offers enhanced diagnostic and prognostic value. Bioinformatics analysis revealed that MMP9 interacts with S100A12, ARG1, SLPI, and ANXA3 proteins. These proteins regulate inflammation and are important indicators of inflammatory conditions. For example, MMP9, as an inflammatory protein, interacts with A8/A9 cells in the S100 family and is involved in thrombosis. However, there is no direct evidence that S100A12 is involved in thrombosis (Gupta et al., 2022). We found that S100A12 plays a crucial role as a key crosstalk gene between SS and VTE, interacts with MMP9, and may indirectly participate in the inflammatory immune response to assist thrombosis. In conclusion, the interaction of MMP9 with these proteins is mainly involved in regulating inflammation and is an important indicator of inflammation status (Zhang et al., 2017). Our results suggest that the functions of MMP9 and its interacting proteins are complex and not limited to the regulation of inflammatory processes but may also play an important role in thrombosis and coagulation regulation.

During functional enrichment analysis, we found that different metabolic pathways were significantly enriched in SS and VTE. Indeed, various metabolic pathways are involved in SS formation and are involved in immune metabolism, metabolic reprogramming, and inflammatory response, and these metabolic processes provide potential therapeutic targets for the study of SS treatment (Xie et al., 2016; Chen et al., 2019). VTE is related to the upregulation of metabolites such as 3-hydroxybutyric acid, aromatic amino acids, and lipids (Prandoni, 2007; Onida et al., 2019), as well as metabolic and immune responses. Elucidating the mechanisms of the metabolic pathways in SS and VTE is extremely complex, presenting challenges and opportunities for further research. Estrogen can trigger endothelial exocytosis leading to vascular thrombosis and inflammation (Kim et al., 2021). The specific role of the estrogen signaling pathway in sepsis and VTE formation warrants further validation by experiments. It has been shown that the FOXO signaling pathway is involved in muscle protein degradation (Crossland et al., 2008), glucose metabolism, and vascular stability during sepsis (Cifarelli et al., 2012). Accordingly, further research should explore the role of this pathway in the formation of SS and VTE. Most reports about these pathways in inflammatory diseases are based on bioinformatics inference, and these data provide new perspectives for the diagnosis and treatment of SS and VTE (Singh et al., 2016; Bouwman et al., 2021).

VTE and SS are considered inflammatory diseases with an immunochromatic background. There is a growing body of evidence that almost all immune cell subtypes and functions are dysregulated in SS. These results reveal important information about immune infiltration of VTE and SS. For example, NK cells, as important immune cells against thrombosis (Wu et al., 2020), are significantly down-regulated in the pathogenesis of SS, which may provide a suitable immune microenvironment for SS to form VTE. We speculate that CD4+ Tem and Macrophages may also have the above function. These provide detailed information for further understanding of the immune mechanism of SS and VTE and possible potential therapeutic targets. It is well-established that M2 macrophages participate in thrombosis events by inhibiting tissue factor expression (Milano et al., 2016). They have been reported to be highly expressed in the vascular region of chronic thromboembolic pulmonary hypertension and may be involved in the pathological process of thrombofibrosis (Bochenek et al., 2017). We also found that in VTE, M2 macrophages also showed a certain correlation with the metabolic pathway and FOXO signaling pathway score, suggesting that M2 macrophages may participate in thrombosis through the above pathways. In this study, we also found that M2 macrophages were significantly negatively correlated with metabolic pathways and FOXO signaling pathway scores in SS patients, suggesting that M2 macrophages may be involved in regulating the above pathological processes and exhibit anti-metabolic phenotypes. However, the mechanism of action of M2 macrophages in SS with VTE and whether the disease can be improved by supplementing M2 macrophages warrant further research.

Class-switched memory B cells were also a common cell type in datasets, and their levels were elevated when metabolic, estrogen, and FOXO signaling pathways were prominently activated in VTE. Current evidence suggests that class-switched memory B cells in sepsis patients are depleted due to adaptive immunity and impaired B cell function, contributing to sepsis-induced immunosuppression (Shankar-Hari et al., 2017; Toro et al., 2020). In the present study, we found that class-switched memory B cells are involved in the pathogenesis of VTE and are close related to the activation of metabolic, estrogen, and FOXO pathways and probably provide a basis for further research on the mechanism of SS with VTE.

In an animal model of sepsis with DVT, we found that MMP9 protein expression was significantly higher than in the control group. GEO database analysis revealed that high expression of MMP9 at the transcriptional and protein levels was associated with sepsis and venous thrombosis, consistent with the literature (Deatrick et al., 2005). Studies using MMP inhibitors and MMP knockout mice have shown that MMPs play a crucial role in infection and host response to infection (Hoffmann et al., 2009). At the same time, thrombus regression was associated with downregulated MMP9 in a mouse model (Terry et al., 2016). Accordingly, directly or indirectly regulating the expression level of MMP9 represents a novel approach to treating venous thrombosis. Indeed, the expression of MMP-9 can exhibit variations across different animal models, tissues, and time points, contributing to discrepancies and controversies regarding its exact role in thrombosis and fibrinolysis (Morishige et al., 2003; Henke et al., 2006). Earlier studies have shown that plasma MMP-9 concentrations may be a useful prognostic marker in septic shock (Nakamura et al., 1998), TIMP-1/MMP-9 ratio is correlated with sepsis severity and coagulation index, and may be a new biomarker of sepsis outcome (Lorente et al., 2014). MMP-9 was also found to be associated with delirium in ICU sepsis (Girard et al., 2012). Due to the cost of testing MMP-9 and the contradiction in a previous report of negative results (Lorente et al., 2009), the promotion and application of MMP-9 in the clinical may be limited. In view of the diversity of MMP9 functions and the complexity of sepsis combined with VTE, how MMP9 is regulated and what specific functions it plays in the pathological model of sepsis combined with VTE need to be further studied in more experiments. In conclusion, our study suggests that MMP9 plays an important role in venous thrombogenesis events in sepsis. MMP9 has huge prospects as a biomarker for diagnosing sepsis with venous thrombosis and a potential molecular target for treatment.

## 5 Conclusion

Metabolic, estrogen, and FOXO pathways play important roles in both SS and VTE and are related to the immune cell microenvironment of M2 macrophages and Class-switched memory B cells. MMP9 shows promise as a biomarker for diagnosing sepsis with venous thrombosis and a potential molecular target for treating this patient population.

## Data availability statement

The datasets presented in this study can be found in online repositories. The names of the repository/repositories and accession number(s) can be found in the article/**Supplementary Material**.

## Ethics statement

The studies involving humans were approved by the Ethics Committee of Tianjin Medical University General Hospital. The studies were conducted in accordance with the local legislation and institutional requirements. The participants provided their written informed consent to participate in this study. The animal studies were approved by the Ethics Committee of Tianjin Medical University General Hospital. The studies were conducted in accordance with the local legislation and institutional requirements. Written informed consent was obtained from the owners for the participation of their animals in this study.

## Author contributions

Conception and design: LD, ZL, CW, XZ. Administrative support: LD. Collection and assembly of data: XX, MW. Data analysis and interpretation: ZL, XZ. Manuscript writing: all authors. Final approval of manuscript: all authors. All authors contributed to the article and approved the submitted version.
