# Supplementary material for: Crosstalk between septic shock and venous thromboembolism: a bioinformatics and immunoassay analysis
Source: Front Cell Infect Microbiol. 2023 Nov 9;13:1235269. doi: 10.3389/fcimb.2023.1235269 (PMC10666789; doi:10.3389/fcimb.2023.1235269)
Supplement: Supplementary file 1 [file Image_1.pdf]

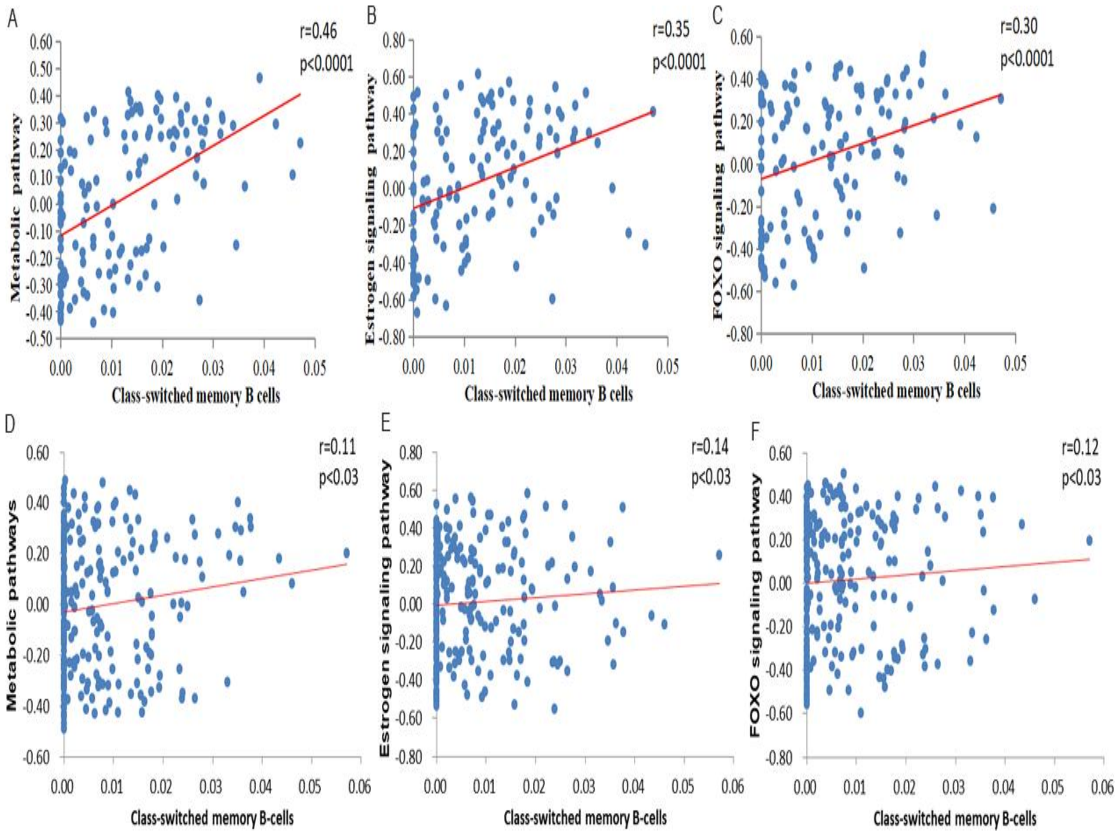

Figure S1. Correlation between key pathways and immune cells. The correlation between class-switched memory B cells and metabolic pathways, estrogen signaling pathway, and FOXO signaling pathway in dataset GSE19151(A 、 B 、 C). The correlation between class-switched memory B cells and metabolic pathways, estrogen signaling pathway, and FOXO signaling pathway is demonstrated in the datasets of SS (D、E、F).
