# Supplementary figures and images for: Crosstalk between septic shock and venous thromboembolism: a bioinformatics and immunoassay analysis
Source: Front Cell Infect Microbiol. 2023 Nov 9;13:1235269. doi: 10.3389/fcimb.2023.1235269 (PMC10666789; doi:10.3389/fcimb.2023.1235269)

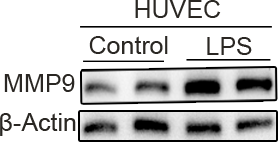

Supplement: Supplementary file 2 [file Image_2.tif]
