# Supplementary material for: Crosstalk between septic shock and venous thromboembolism: a bioinformatics and immunoassay analysis
Source: Front Cell Infect Microbiol. 2023 Nov 9;13:1235269. doi: 10.3389/fcimb.2023.1235269 (PMC10666789; doi:10.3389/fcimb.2023.1235269)
Supplement: Supplementary file 3 [file Table_1.pdf]

	Normal	SS	*p* value
Cases	10	12	
Gender [male (male %)]	6(60%)	8(67%)	
Age(years)	35.9±7.87	75±11.23	<0.0001
Body mass index	24.09±2.00	25.69±7.21	<0.0001
White blood cell count	9.16±2.41	15.81±4.33	0.0003
IL-6 (pg/ml)	6.52±2.74	152.35±188.62	0.0003
PCT (ng/ml)	0.04±0.03	14.06±11.98	0.0015
D-dimer (ng/ml)	323.2±130.46	4400.67±2615.51	<0.0001
