# Supplementary material for: Crosstalk between septic shock and venous thromboembolism: a bioinformatics and immunoassay analysis
Source: Front Cell Infect Microbiol. 2023 Nov 9;13:1235269. doi: 10.3389/fcimb.2023.1235269 (PMC10666789; doi:10.3389/fcimb.2023.1235269)
Supplement: Supplementary file 4 [file Table_2.pdf]

Gene	Species	Direction	Primer sequence 5'→3'
MMP9	Human	Forward	GGCACCACCACAACATCACCTA
		Reverse	CGGGCAAAGGCGTCGTCAAT
BCL2A1	Human	Forward	TACAGGCTGGCTCAGGACTAT
		Reverse	CGCAACATTTTGTAGCACTCTG
S100A12	Human	Forward	TCCACCAATACTCAGTTCGGAAG
		Reverse	ACTCTTTGTGGGTGTGGTAATGG
RNASE2	Human	Forward	TGTGGTAACCCAAATATGACCTG
		Reverse	GGTCTCGTCGTTGATCTCTGT
ARG1	Human	Forward	TGATGTTGACGGACTGGACC
		Reverse	ATCTAATCCTGAGAGTAGCCCTGT
MS4A4A	Human	Forward	TGAGCCTTAGCATGGGAATAACA
		Reverse	CCCGATATACACGGAAATAGGGT
SLPI	Human	Forward	AATGCCTGGATCCTGTTGAC
		Reverse	AAAGGACCTGGACCACACAG
ANXA3	Human	Forward	CCTTCGCTCGCAGTTTGTTC
		Reverse	TCGGTGTCCAACCCAGATAGA
