# Supplementary material for: Crosstalk between septic shock and venous thromboembolism: a bioinformatics and immunoassay analysis
Source: Front Cell Infect Microbiol. 2023 Nov 9;13:1235269. doi: 10.3389/fcimb.2023.1235269 (PMC10666789; doi:10.3389/fcimb.2023.1235269)
Supplement: Supplementary file 5 [file Table_3.pdf]

Data Table S3 General characteristics of follow-up in patients with septic shock and VTE.

Basic information		Survival（n=19）	Death (n=17)	*p*
Age(year)		72.53±10.01	74.18±10.04	0.63
Gender (n, %)	Female	12(63.16)	7(41.18)	0.19
	Male	7(36.84)	10(58.82)	
Body mass index (kg/m2)		26±5.55	24.72±3.21	0.41
White blood cell count (×109)		13.26±4.68	12.73±4.34	0.73
D-dimer (ng/ml)		3004.32±1834.70	2530.06±1491.41	0.34
Prothrombin time (sec)		12.58±2.82	19.78±18.59	0.1
International normalized ratio		1.19±0.25	1.86±1.62	0.09
Activated partial thromboplastin time		33.1±7.42	38.14±21.65	0.35
Thrombin time (sec)		16.56±3.51	23.51±20.11	0.15
Fibrinogen (g/L)		3.46±0.64	3.54±1.39	0.81
Total bilirubin (umol/L)		19.45±6.78	19.25±9.67	0.94
IL-6 (pg/ml)		161.96±200.79	96.98±71.12	0.22
PCT (ng/ml)		6.6±10.7	6.45±10.57	0.97
Smoking history (n, %)	No	9	9	0.74
	Yes	10	8	
Drinking history (n, %)	No	12	7	0.19
	Yes	7	10	
Hypertension (n, %)	No	5	8	0.2
	Yes	14	9	
Diabetes (n, %)	No	8	8	0.77
	Yes	11	9	
